# Characteristics of the *BMP7* Promoter in Hu Sheep

**DOI:** 10.3390/ani9110874

**Published:** 2019-10-28

**Authors:** Xiaoyang Lv, Wei Sun, Shuangxia Zou, Ling Chen, Joram M. Mwacharo, Jinyu Wang

**Affiliations:** 1 College of Animal Science and Technology, Yangzhou University, Yangzhou 225009, China; dx120170085@yzu.edu.cn (X.L.); zshx201422@163.com (S.Z.); 2 Joint International Research Laboratory of Agriculture and Agri-Product Safety of Ministry of Education of China, Yangzhou University, Yangzhou 225009, China; 3 Animal Science and Veterinary Medicine Bureau of Suzhou City, Suzhou 215200, China; zhongyc8084@163.com; 4 Suzhou Stud Farm, Suzhou 215200, China; 5 Small Ruminant Genomics Group, International Center for Agricultural Research in the Dry Areas (ICARDA), Addis Ababa P.O. BOX 5689, Ethiopia; j.mwacharo@cgiar.org

**Keywords:** Hu sheep, *BMP7*, promoter, transcription factor

## Abstract

**Simple Summary:**

Bone morphogenetic protein 7 (BMP7) is one of the largest secretory signal conductive molecules and is in the TGF-β superfamily. It plays an important role in the growth and development of hair follicles. We cloned the proximal promoter of the *BMP7* gene for bioinformatics analysis. Dual-luciferase reporter system and overexpression were used to analyze the key regions and transcription factor binding sites. There was high activity between −758 bp and −545 bp in the core region of the gene and a possible binding site for transcription factors *SP1* and *EGR1*.

**Abstract:**

The *BMP7* gene is involved in the growth and development of hair follicles but its regulation mechanism is unclear. We studied the regulation mechanism of the *BMP7* promoter by cloning the proximal promoter of *BMP7* for bioinformatics analysis. A series of missing vectors was then constructed for dual-fluorescein activity detection based on the bioinformatics analysis results. We tested transcription-factor binding-site mutations and transcription factor over-expression to analyze the transcriptional regulation principle of the *BMP7* promoter region. The upstream transcriptional regulatory region of the *BMP7* gene proximal promoter was predicted by bioinformatics. There were −1216 bp to −1166 bp and −632 bp to −582 bp transcription initiation sites in the upstream transcriptional regulatory region of the *BMP7* gene proximal promoter. The CpG islands’ distribution showed that there were many CpG islands at −549 bp to 1 bp. A dual-luciferase assay revealed high activity between −758 bp and −545 bp in the core region and a possible binding site for transcription factors *SP1* and *EGR1*. The transcriptional activity of *BMP7* was significantly decreased in the transcriptional regulatory region of the *BMP7* after *EGR1* and *SP1* mutation. Transcription was significantly enhanced by over expression of the *EGR1* transcription factor, which strongly suggests that *EGR1* and *SP1* play important roles in *BMP7* regulation.

## 1. Introduction

Bone morphogenetic protein (BMP) belongs to the transforming growth factor-β (TGF-β) superfamily [1]. The BMP family has many biological functions in cell proliferation, differentiation, bone formation, tumors, and hair follicle growth. The BMP signaling pathway may inhibit the Wnt/β-catenin signaling pathways to promote hair cycle growth [2] and enable the hair follicle stem cells in the growth cycle to be stationary [3]. The BMP signaling pathway is critical for the differentiation of hair root sheaths and hair shafts. Kulessa [4] found that transgenic mice overexpressed the BMP antagonist noggin caused hair matrix cells proliferation and hair precursor cells differentiation leading to hair growth. In noggin-knockout mice, prolonged periods of excessive BMPs impeded the development of primary hair follicles and affected the formation process of inner root sheaths and hair shafts [5,6]. In addition, Noramly [7] found that BMP7 is related to the size and spatial distribution of feather embryos. These studies identified BMPs as regulators of the genetic control of hair shaft differentiation in hair follicles. The BMP7 is one of the most biologically active proteins in the BMP family and it is also an important signaling molecule involved in hair follicle development. The *BMP7* gene is highly expressed when hair follicles are in their growth phase, especially when the cells begin to proliferate at the basement membrane. When hair follicle enters the degenerative phase or the resting phase, *BMP7* is low or undetected [8]. Wang [9] noted that *BMP7* can inhibit wool growth in fine wool sheep. These results indicate that *BMP7* may promote the proliferation of hair follicle cells in the hair follicle growth phase.

High-throughput sequencing allows the screening of trait-related genes. Research on the regulation of gene expression is a current research emphasis area. Research on small RNA is another research focus, but promoters are also key components of the expression regulation. Understanding the promoter structure is the first step in determining transcriptional regulation. The eukaryotic promoter is located upstream of the 5′ end of the gene and it can bind to RNA polymerase II to regulate gene transcription. This regulation affects cell growth, differentiation, and apoptosis and is the main method of regulating gene expression. To clarify the molecular mechanism of gene transcription regulation, understanding the transcriptional regulation of the promoter is necessary. In this study, we cloned the proximal promoter region of *BMP7* and predicted the transcription initiation site and CpG island (a 200 bp region of DNA with a high G_C content greater than 50% and observed CpG_expected CpG ratio of greater or equal to 0.6). A series of deletion vectors were constructed to determine the core region using a dual-luciferase assay. Based on the core region, we analyzed the transcription factor sites by bioinformatics analysis methods. Site-directed mutagenesis and transcription factor over-expression tests were performed to verify the authenticity of the candidate *BMP7* transcription factor. Finally, we identified the transcription factor of *BMP7*.

## 2. Material and Methods

### 2.1. Ethics Statement

The Institutional Animal Care and Use Committee (IACUC) of the government of Jiangsu Province (Permit No. 45) and the Ministry of Agriculture of China (Permit No. 39) approved the animal study proposal. All experimental procedures were conducted in strict compliance with the recommendations of the Guide for the Care and Use of Laboratory Animals of Jiangsu Province and of the Animal Care and Use Committee of the Chinese Ministry of Agriculture. Efforts were made to minimize animal suffering.

### 2.2. Sample Collection

Sheep were obtained from the Suzhou stud farm in China. A 0.5 cm^2^ sample of ear tissue was collected from each sheep. Tissue was frozen with liquid nitrogen and ground to a powder. An animal Tissues/Cells Genomic DNA Extraction Kit (Solarbio, Beijing, China) was used to extract DNA from the ground ear tissue. Extracted DNA was stored at −20 °C.

### 2.3. Bioinformatics Analysis of Proximal Promoter

According to the *BMP7* promoter sequence, bioinformatics software and websites were used to predict basic *BMP7* information. Promoter 2.0 Prediction Server [10] and NNPP [11] were used to predict the core region of the proximal promoter of *BMP7*. MethPrimer [12] was used to predict CpG islands. TRANSFAC [13] and JASPAR [14] were used to predict the transcription factor binding site.

### 2.4. Deletion Plasmids Construction

According to the DNA sequence in NCBI (Gene ID: 443175), Oligo7 was used to design different primers for deletion fragments to amplify the BMP7 promoter region. The restriction sites (KpnI and HindIII) were added to the 5′ ends of the forward and reverse primers. The volume of the PCR amplification system was 50 μL, including 25 μL of 2 × GC Buffer I (II) (Takara, Dalian, China), 8 μL of dNTP Mix (Takara, Dalian, China), 2 μL of 10 mmol/L primer (forward and reverse), 0.5 μL of LA Taq (Takara, Dalian, China), 2 μL of DNA, and 10.5 μL of ddH_2_O. The amplification program was as follows: initial denaturation at 94 °C for 1 min, followed by an optimal number of cycles (33 cycles) of denaturation at 94 °C for 30 s, annealing at 68 °C for 30 s, extension at 72 °C for 60 s, with a final extension at 72 °C for 5 min. The final PCR product was stored at 4 °C.

MiniEST Agarose Gel DNA Extraction Kit (TaKaRa, Dalian, China) was used to recover the PCR product. Connection system: Recycling PCR product 4 μL, pGL3-Base vector 1 μL, Solution I 5 μL, connection at 16 °C for 16 h; 2 μL ligation product was added to a 50 μL of DH5α competent cell and suspended on ice for 30 min at 42 °C for 45–60 s; after 2 min, ice bath Liquid LB medium was added (948 μL), and the mix was shaken with 120 rpm at 37 °C for 1 h. Then, 50 μL of LB medium with DH5α was applied on the LB plates with Amp culturing at 37 °C for 12–16 h. A single colony was selected for culturing and plasmid purification. Polymerase Chain Reaction(PCR) and enzyme digestion experiments were used to perform colony selection. The positive plasmids were sent to Sangon Biotech Co. Ltd. (Shanghai, China) for sequencing. The EndoFree Maxi Plasmid Kit (DP117) (TaKaRa, Dalian, China) was used to extract the plasmids and they were stored at −20 °C. We selected the sequence from 2000 bp upstream and 500 bp downstream of the initiation codon ATG of *BMP7* (−2000 bp to +500 bp), and different fragments of the promoter region were amplified, including B1(−1500 bp to +257 bp), B2 (−985 bp to +29 bp), B3 (−758 bp to +29 bp), and B4 (−545 bp to +29 bp). Then, we constructed B5 (−758 bp to −545 bp) fragment vectors for regions with high transcriptional activity (Table 1).

### 2.5. Transfection and Dual-Luciferase Assay

The HEK293T cells were cultured in a 24 well plate at a density of 5 × 10^5^ with DMEM/F12 (Gibco, New York, NY, USA) supplemented with 10% FBS (Sigma, San Francisco, CA, USA) with 5% CO2 in air at 37 °C. After 24 h, the cells were cultured to 80% confluence and transfected using FuGENE^®^ HD Transfection Reagent (Promega, WI, USA), according to its manufacturer instructions. The target plasmid and the internal reference plasmid pGL-TK were co-transfected into the cells at a ratio of 15–20:1, and negative and positive controls were set. After 24 h, 10 μL of cell lysate were added to the non-transparent 96 well plate, and 50 μL of Luciferase Reagent were added to immediately measure the Firefly luciferase activity with the Synergy 2 chemiluminescence detector (Bio Tek, Vermont, USA). The corresponding Renilla luciferase activity values were measured immediately after adding 50 μL of 1× Stop & Glo^®^ Reagent.

### 2.6. Mutant and Overexpression Plasmids Construction and Dual-Luciferase Assays

Bioinformatics analysis of the regions with high transcriptional activity revealed two transcription factors *EGR1* and *SP1* with high scores. To verify whether these two transcription factors have significant effects on transcription activity, transcription factor binding sites were mutated. According to the primer design requirements of the Fast Site-Directed Mutagenesis Kit (Tiangen, Beijing), the transcription factor point mutation primers (Table 2) were designed using Oligo7. The mutation plasmid BT1 (mutation of *EGR1* binding site) and BT2 (mutation of *SP1* binding site) were constructed using the B5 fragment as a template, respectively. The *EGR1* overexpression plasmid was constructed by Suzhou Jinglun Biotechnology Co., Ltd., Suzhou, China.

### 2.7. Statistical Analysis

The relative activity of luciferase (the ratio of firefly luciferase activity value to Renilla luciferase activity value) was analyzed using one-way analysis of variance (ANOVA). A *p* < 0.05 was considered to be a significant. The statistics software used was SPSS version 16.0.

## 3. Results

### 3.1. Bioinformatics Analysis of the Proximal Promoter Region of Hu Sheep *BMP7*

**Figure 1:**
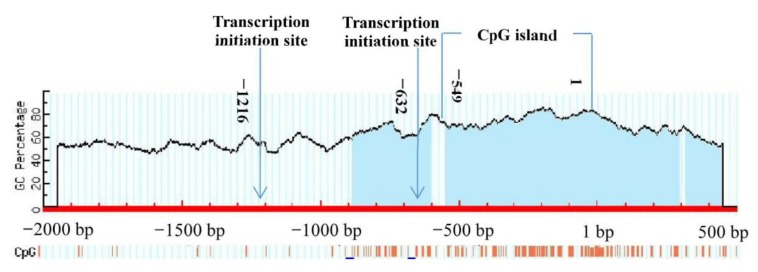
A region of DNA with a high G_C content (greater than 50%) and observed CpG_expected CpG ratio of greater or equal to 0.6 (CpG islands) predicted in the bone morphogenetic protein 7 (*BMP7*) promoter. Note that the site of “1 bp” means the initiation codon (ATG). The −2000 bp indicator means 2000 bp in the upstream region of the transcription start site, and the 500 bp indicator means 500 bp in the downstream region of the transcription start site.

The 2000 bp upstream sequence and the 500 bp downstream sequence of the *BMP7* from translation initiation codon (ATG) were retrieved from the NCBI database. We predicted the possible transcription initiation site. The results showed that there were two possible locations including −1216 bp to −1166 bp and −632 bp to −582 bp. The CpG island prediction of the *BMP7* promoter was performed using CpG finder and MethPrimer. The prediction was based on Island size > 100, GC Percent > 50.0, Obs/Exp > 0.6. The results showed CpG-rich islands at −549 bp to 1 bp, with a fragment length of 549 bp (Figure 1).

### 3.2. Deletion Fragment Analysis of the *BMP7* Promoter

**Figure 2:**
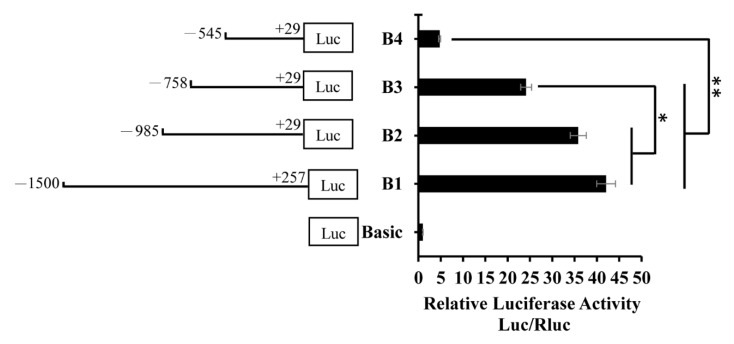
Relative luciferase activity of the Hu sheep *BMP7* promoter with different lengths. Note that the basic was the pGL3-Base vector. For B1, the target fragment (−1500 bp to +257 bp) was ligated to the pGL3-Base vector; for B2, the target fragment (−985 bp to +29 bp) was ligated to the pGL3-Base vector; for B3, the target fragment (−758 bp to +29 bp) was ligated to the pGL3-Base vector; and for B4, the target fragment (−545 bp to +29 bp) was ligated to the pGL3-Base vector. The basic was the blank control group and B1 was the normal control group. The marker “*” represents a significant difference (*p* < 0.05), while “**” represents a highly significant difference (*p* < 0.01).

**Figure 3:**
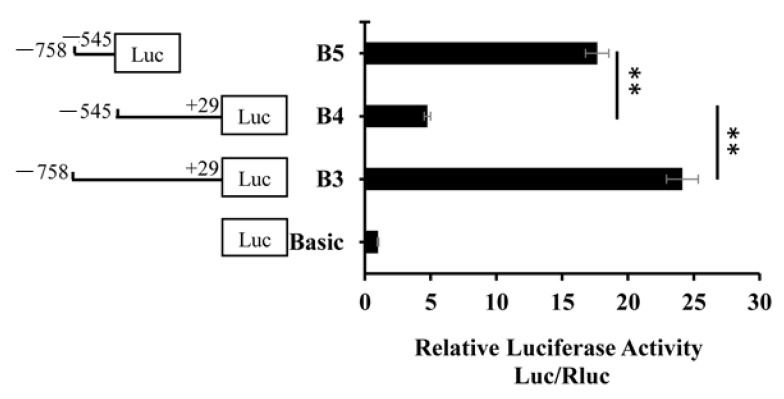
Activity analysis of the −758 bp to −545 bp region of the Hu sheep *BMP7* promoter. Note that for B5, the target fragment (−758 bp to −545 bp) was ligated to the pGL3-Base vector; B3 was the normal control group. The marker “**” represents a highly significant difference (*p* < 0.01).

In order to identify the core region of the *BMP7* promoter, four deletion plasmids were constructed. They were used to transfect cells and detect dual-luciferase activity (Figure 2). The activity of B1 and B2 fragments were similar and the difference was not significant. Compared with B1 and B2, the activity of B3 significantly decreased (*p* < 0.05). Compared to B1, B2, and B3, the activity of B4 significantly decreased (*p* < 0.01). The promoter activity was shown between B3 and B4, specifically in the −758 bp to −545 bp region. The total activity of B4 and the −758 bp to −545 bp region was not different than that of the B3 (Figure 3). Therefore, the −758 bp to −545 bp region may have a cis element that positively regulates the *BMP7* promoter activity.

### 3.3. Conservative Analysis of Transcription Core and Prediction of Transcription Factors

**Figure 4:**
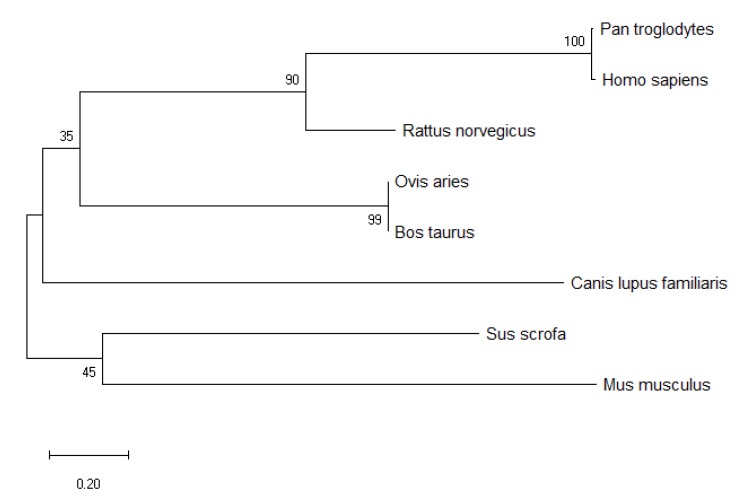
*BMP7* promoter sequence (−758 to −545 bp) phylogenetic tree of eight species.

To identify the conservation of the transcriptional core sequences (−758 to −545 bp), MEGA X software was used for homology analysis between the transcriptional core sequences of *BMP7* and the published sequences in Genbank *Homo sapiens* (NM_001719.3), *Mus musculus* (NM_007557.3), *Rattus norvegicus* (NM_001191856.2), *Sus scrofa* (NM_001105290.1), *Bos taurus* (NM_001206015.1), *Canis lupus familiaris* (NM_001197052.1), *Pan troglodytes* (XM_001170064), *Canis lupus familiaris* (NM_001197052.1); we then constructed a phylogenetic tree (Figure 4). Homology analysis showed that: the transcriptional core sequences of the Hu sheep *BMP7* promoter had the highest homology (99%) with *Bos taurus*. However, the promoter transcriptional core sequences of sheep and other species (*Homo sapiens*, *Pan troglodytes*, *Rattus norvegicus*, *Canis lupus familiaris*, *Sus scrofa*, and *Mus musculus*) have very low homology. Sequence analysis showed that homology of the transcriptional core sequences (−758 to −545 bp) between sheep and other species was low, except for *Bos Taurus*.

We screened for possible transcription factors and JASPAR was used to predict the transcription factor between −758 and −545 bp of the *BMP7* promoter. There were no typical RNA polymerase II binding elements, such as CAAT and TATA boxes. In the prediction results, ZNF263 had the highest score, and SP1 had the highest frequency of occurrence. The possible transcription factors in this region also included *EGR1*, *NRF1*, *KLF5, FOXI1*, *FOXB1*, and *FOXC1*. Analysis of the prediction results showed that the transcription factors *SP1* and *EGR1* had higher scores, and they were involved in cell growth and development. We selected these factors for verification and analysis and believe that they play important roles in transcriptional regulation (Table 3).

### 3.4. Verification of Point Mutation of Transcription Factor

**Figure 5:**
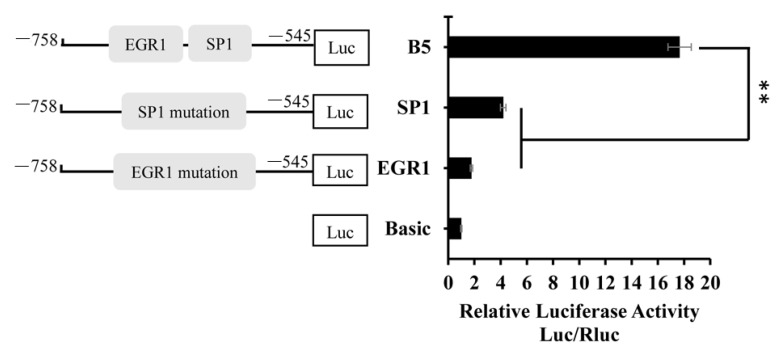
Point mutation analysis of *EGR1* and *SP1.* Note that the binding sites of *EGR1* and *SP1* were in the B5 fragment. *SP1*: the *SP1* mutation. *EGR1*: the *EGR1* mutation. The marker “**” represents a highly significant difference (*p* < 0.01).

The transcription activity of the B5 (−758 to −545 bp) fragment is high. Transcription factors *EGR1* and *SP1* exist in this fragment and these two transcription factors have an effect on the transcription activity. Therefore, we determined whether the transcription activity of the B5 fragment is reduced by site-specific mutations of *EGR1* and *SP1*, respectively. Compared to B5 fragment activity, we analyzed the transcription activity level after mutation. Compared with the non-mutated groups, the promoter activity significantly decreased (*p* < 0.01) after *EGR1* and *SP1* mutations, and there was a particularly more significant difference between the B5 and the *EGR1* mutant plasmids (Figure 5). Therefore, these two transcription factors *EGR1* and SP1 may play an important role in the regulation of promoter activity; as *EGR1* had a greater influence on *BMP7* promoter activity, an *EGR1* overexpression plasmid was constructed to further verify the binding sites.

### 3.5. Overexpression of *EGR1* on Transcriptional Activity of the *BMP7* Promoter

**Figure 6:**
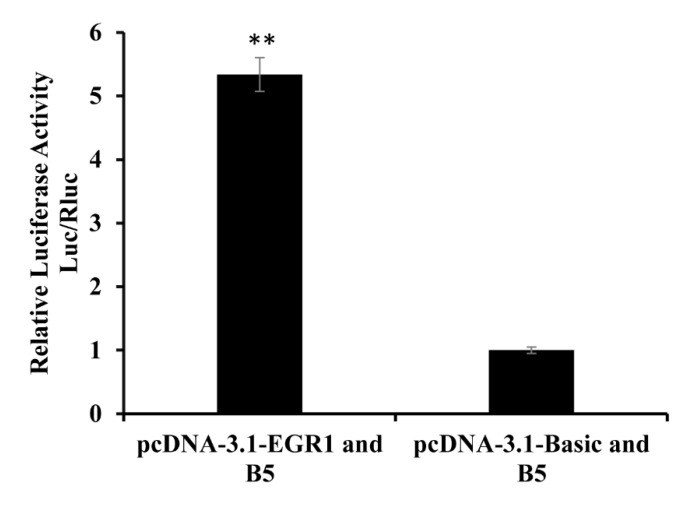
Relative activity analysis of *EGR1*. Note that for pcDNA-3.1-*EGR1* and B5, the *EGR1* overexpression vector was co-transfected with the B5 fragment; for pcDNA-3.1-Basic and B5, the pcDNA-3.1 vector was co-transfected with the B5 fragment. The group of pcDNA-3.1-Basic and B5 was the control group. The marker “**” represents a highly significant difference (*p* < 0.01).

To study binding sites of the *EGR1* transcription factor in the *BMP7* promoter, the *EGR1* overexpression plasmid was co-transfected with the B5. The dual-luciferase activity in the overexpression group was significantly higher (*p* < 0.01) than in the control group (Figure 6). Overexpression of *EGR1* may have affected the transcriptional activity of B5. The *EGR1* transcription factor binding site may exist in the *BMP7* promoter region. This could affect the transcriptional activity and regulation of the *BMP7* promoter.

## 4. Discussion

### 4.1. Characteristics of the Hu Sheep *BMP7* Promoter

Among molecular regulatory processes, transcription regulation has a significant impact on gene expression. The gene *BMP7* is well known to be expressed in bone, kidney, and skin [15], and it has many biological functions, including participation in bone formation and regeneration, hair follicle growth and development, and the growth and development of the reproductive system. [6,16,17]. However, there is little research on the transcriptional regulation of *BMP7*. It is necessary to study the regulation of *BMP7* at the transcriptional level because the promoter is the most important transcriptional regulatory element. We analyzed the promoter activity of the 5′-flanking region of *BMP7* and found that a positive regulatory region is located upstream of the initiation codon (−758 bp to −545 bp). Similar to our results, the tongue sole *BMP7* promoter contained one potential region located upstream of the initiation codon (−740 bp to −396 bp) [18]. However, there are multiple core transcription regions (−3070 bp to −1771 bp; −1277 bp to −491 bp) in the human *BMP7* promoter [19]. In addition, Simon [20] found that the sequence within the 1394 bp *BMP7* 5′-flanking region has a high activity. We found that the sequence within 1500 bp *BMP7* 5′-flanking region has a high activity in human and tongue sole. The Hu sheep *BMP7* promoter contained two main transcription initiation sites (−1216 bp and −632 bp). The human *BMP7* promoter contains a single transcription initiation site (−764 bp) [19].

### 4.2. The Regulatory Elements Involved in Control of Hu Sheep *BMP7* Expression

Gene transcriptional regulation by a promoter occurs in a system in which the transcription factor and regulatory elements are the most important components [21]. There are many regulatory elements and transcription factor binding sites on each promoter, such as CAAT box, TATA box, *SP1*, and *AP1*. Transcription factors are trans-acting factors that are located on promoter sequences and bind to cis-acting elements to regulate transcriptional regulation [22]. In the present study, regulatory elements and transcription factors were identified. There were no typical RNA polymerase II binding elements (CAAT and TATA box) in the positive regulatory region (−758 bp to −545 bp). No TATA and CAAT boxes were found in the positive regulatory region of *BMP7* in mice [20]. Previous studies have detected that only 10–20% of mammalian promoters had TATA box. The transcriptional regulation processes of mammals can be performed without the TATA box [23,24].

We predicted transcription factor binding sites were in the core region of the promoter of *BMP7*, and we screened transcription factors related to transcriptional regulation. The transcription factors *SP1*, *SP2*, *ZNF263*, *EGR1*, *NRF1*, *KLF5*, *FOXI1*, *FOXB1*, and *FOXC1* were predicted from −758 to −545 bp of the Hu sheep *BMP7* promoter. At present, some reports showed that SNPs on promoters could affect the binding of transcription factors. Four SNPs were found on a porcine *BMP7* promoter, including T-3722C, A-3684G, C-3522G, A-1027G. T-3722C, C-3522G, and A-1027G were located at transcription factor binding site and affected the transcriptional activity [25]. Therefore, site-directed mutagenesis experiment is needed to study the transcription factors. In our study, transcription factors *EGR1* and *SP1* with high scores were selected by site-directed mutagenesis and transcription factor overexpression experiments. The *SP1* has also been in the *BMP7* promoter of other mammal species [19,20], while *EGR1* appeared in the *BMP7* promoter core region of Hu sheep. We speculate that *EGR1* might participate in the transcriptional regulation of *BMP7*.

### 4.3. Functions of *EGR1* and *SP1*

The transcription factors *SP1* and *SP2,* belong to the SP/KLF family. It is known that *SP1* binds to the GC/CT cassette in the promoter region of the gene, activates the transcription process, and is abnormally expressed in various tumor groups [26,27,28]. The DNA binding activity of *SP1* in human dermal papilloma was significantly higher than that of normal cells [29]. In addition, *SP2* has both activation and inhibitory effects; it competes with *SP1* or *SP4* to bind to the GC/CT cassette, thereby inhibiting the onset of transcription [30,31]. Early growth response protein 1 (*EGR1*) belongs to the immediate early gene family. It is an important transcription factor that activates or inhibits the transcription of genes and, thus, participates in the regulation of cell proliferation and cell apoptosis, growth, and differentiation [32,33]. Kwon [34] found that patulin *HCT116* promoters promote the *EGR1* binding promoter and inhibits cell growth. Kruppel-like factor 5 (*KLF5*) is a family of Sp/Kruppel transcription factors that regulates transcription by binding to a promoter. These transcription factors are abundantly expressed in epithelial tissues and are involved in cell proliferation, embryonic development, and tumorigenesis [35,36,37,38].

## 5. Conclusions

**Figure 7:**
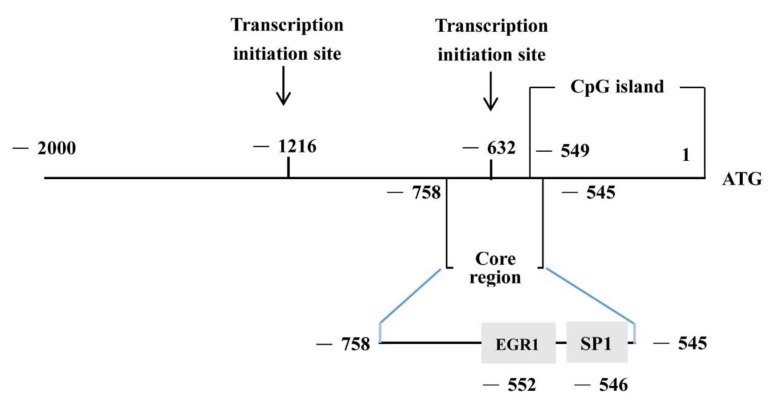
Schematic diagram of the *BMP7* promoter structure of from Hu sheep.

This study found a high degree of activity in the *BMP7* promoter region between −758 bp and −545 bp and its homology between sheep and other species was low, except for *Bos Taurus*, and CpG-rich islands at −549 bp to 1 bp, with a fragment length of 549 bp. The transcription factors *SP1* and *EGR1* may have binding sites that regulate the *BMP7* gene promoter. Both *EGR1* and *SP1* could be used as candidate transcription factors for future studies (Figure 7).

## Figures and Tables

**Table 1:** Primers for deletion plasmids construction from the Hu sheep *BMP7* promoter.

Name	Sequence (5′–3′)	Product Length/bp
B1	F: GG**GGTACC**CCAAGTGGGCAACTCAGTGTC	1757
	R: CC**AAGCTT**GTACAGGTCCAGCATGAACATGG	
B2	F: GG**GGTACC**CAGTACAAAGGCAAACTGGCAACA	1014
B3	F: GG**GGTACC**CGCTCGTATTCCCCTCTCCGCATC	787
B4	F: GG**GGTACC**CCCAGGCCCCAGCGCGTACCA	574
	R: CC**AAGCTT**CCAGAGCGCCACGAAGCTGT	
B5	F: GG**GGTACC**CGAGTCCGGAGAAGGCAGG	214
	R: CC**AAGCTT**GCTGGGGCCTGGGAGGAGGA	

Note that the underline represents enzyme sites. B2, B3, and B4 have the same reverse primer.

**Table 2:** Site-directed mutation primers of the Hu sheep *BMP7* promoter.

Name	Primer Sequence (5′–3′)	Length/bp
BTF1	GCCCTTGGAAAGGCCGTCCTC**TTCT**CCCTCCTCCTCCCAG	214
BTR1	CTGGGAGGAGGAGGGAG**AAGA**GGACGGCCTTTCCAAGGGC	
BTF2	AGGCCGTCCTCCTCCCCC**GCCGCCG**CCCAGGCCCCAGC	214
BTR2	GCTGGGGCCTGGG**CGGCGGC**GGGGGAGGAGGACGGCCT	

Note that the underline means mutation sites.

**Table 3:** Transcription factor binding sites predicted in the region from −758 bp to −545 bp of *BMP7* promoter.

Region	Transcription Factor	Start Site	Sequence
−758 bp to −545 bp	*FOXB1*	−675 (1)	TCAGTAAATAT
	*FOXC1*	−675 (1)	TCAGTAAATAT
	*FOXC2*	−675 (1)	TCAGTAAATATT
	*FOXD2*	−672 (1)	GTAAATA
	*FOXI1*	−672 (1)	GTAAATA
	*FOXL1*	−672 (1)	GTAAATA
	*FOXO4*	−672 (1)	GTAAATA
	*Klf4*	−612 (1)	AGGGCGGGGC
	*NRF1*	−552 (1)	CCGCGGGCGCG
	*EGR1*	−552 (1)	CCTCCTCCCCCTCC
	*KLF5*	−552 (1)	CCTCCTCCCC
	*SP1*	−552 (1)	CCTCCTCCCCC
	*EGR1*	−546 (1)	CCCCCTCCTCCTCC
	*SP1*	−546 (1)	CCCCCTCCTCC
	*SP2*	−546 (1)	CCCCCTCCTCCTCCC
	*SP1*	−546 (1)	CCTCCTCCTCC

Note that this table shows the cis elements appearing in JASPAR programs. The score was more than 10 and the relative score was more than 0.9.
